# Developing pharmacist practice through a pilot family practice preceptorship programme: a prospective quantitative pre–post evaluation

**DOI:** 10.1007/s11096-026-02198-w

**Published:** 2026-08-06

**Authors:** Tamara M. Cairney, Heather Harrison, Mairi-Anne McLean, Tania Ramos, David Kernaghan, Chris F. Johnson

**Affiliations:** 1 Pharmacy Services, NHS Greater Glasgow and Clyde, Clarkston Court, 56 Busby Road, Glasgow, Scotland G76 7AT UK; 2 Pharmacy Services, Glasgow City Health and Social Care Partnership (North East Locality), NHS Greater Glasgow and Clyde, Glasgow, G69 6GA UK; 3 School of Health in Social Science, University of Edinburgh, Teviot Place, Edinburgh, EH8 9AG UK

**Keywords:** Non-medical prescribing, Pharmacists, Preceptorship, Primary health care

## Abstract

**Introduction:**

Primary care health services face workforce pressures internationally. Greater multidisciplinary working is seen as a solution. In Scotland, pharmacists are embedded within primary care teams. General Practice Clinical Pharmacists (GPCPs) deliver medicines-related services, including independent prescribing, medicines optimisation and medication review. However, GPCPs’ clinical experience and training vary considerably. A structured preceptorship programme was developed to support role development.

**Aim:**

To evaluate the impact of a preceptorship programme for GPCPs on self-assessed training needs, competence and confidence within routine primary care practice.

**Method:**

A prospective repeated-measures survey and medication review activity analysis were conducted with GPCPs in one health board between January 2024 and March 2025. Reported using the Checklist for Reporting of Survey Studies (CROSS). Preceptees, identified using eligibility criteria, completed a 32-item Hennessy-Hicks Training Needs Analysis questionnaire pre- and post a 12-week preceptorship programme, rating tasks for importance, self-perceived performance and confidence. Matched pre-post questionnaire responses were analysed using the Wilcoxon signed-rank test. Routinely recorded medication review activity was extracted from electronic clinical systems for six months pre- and post-programme and summarised descriptively.

**Results:**

Fifty-three GPCPs enrolled; 51 (96%) completed (median age 34 [range 24–57] years; 43 (84%) female. Median years qualified were 11 (range 1–33) and 3 years (0–10) primary care experience. Pre- to post-response rate was 76% (39/51). At baseline, the top five scoring self-reported priority training needs items (task importance minus perceived performance score) of the 32 items were: mental health and chronic pain reviews, conflict management, biopsychosocial assessment, and assessing patient decision-making capacity. At 12 weeks all scores had reduced, with larger reductions observed in the top five items (*p* < 0.001 for each item). Self-reported competence and confidence across all superordinate categories appeared to increase. Recorded medication review activity was summarised descriptively. Median monthly reviews increased from 2 (IQR 0–4) before preceptorship to 4 (IQR 1–10) during preceptorship, before returning to 2 (IQR 0–7) after programme completion.

**Conclusion:**

Structured preceptorship was associated with improved self-reported competence and confidence and a short-term increase in recorded medication review activity. Further evaluation is required to determine whether these changes translate into sustained clinical activity for improved patient outcomes.

**Supplementary Information:**

The online version contains supplementary material available at https://doi.org/10.1007/s11096-026-02198-w.

## Impact statements

- This evaluation provides evidence that structured preceptorship may help pharmacists in patient-facing primary care roles to identify and address perceived development needs.
- The findings indicate clinical areas where pharmacists developing advanced primary care practice may require targeted support such as mental health and chronic pain management, conflict management, biopsychosocial assessment and capacity-related decision making.
- Future pharmacist workforce developments should consider embedding structured supervision, feedback and evaluation of patient-facing activity with a focus on prescribing quality and patient outcomes.

## Introduction

Primary care and family medicine services are experiencing workforce pressures [1–4], driven by physician shortages, workload transfer, aging populations and multimorbidity [5, 6]. Integrating pharmacists, known as General Practice Clinical Pharmacists (GPCPs) in the United Kingdom (UK), into primary care multidisciplinary teams can release family physician capacity by undertaking medicines-related activities, including independent prescribing, medication review and supporting other healthcare professionals with prescribing by addressing complex prescribing issues and optimising patient care in the UK, North America and Australasia [7–13].

The UK Royal College of Pharmacy (RCPharm) and Royal College of General Practitioners endorse integration to relieve service pressures and improve patient care [14]. Therefore, over the past decade, the general practice (family medicine) pharmacy workforce has increased [15, 16] with many transitioning from community and hospital pharmacy roles. In Scotland, since 2018, the Pharmacotherapy Service within the national family medical services contract has promoted greater pharmacy integration to release family physician capacity [17], and futher National Health Service (NHS) Scotland’s aims to reduce inappropriate prescribing, medicines-related harms and improve cost-effective prescribing [18]. To minimise variations in practice, NHS Education Scotland developed training programmes to bridge skills gaps [19]. However, the 12-month COVID-19 lockdown period possibly hindered GPCP professional development due to increased workloads, professional isolation and limited training opportunities [20].

Despite policy initiatives and the growing presence of GPCPs in primary care settings, training gaps exist for pharmacists transitioning into, or developing their role, including cardiology and mental health management [21–23]. Some GPCPs may lack sufficient examination skills, clinical experience and knowledge of integrated working to be effective in their roles [22, 24]. Completion of the RCPharm Core Advanced framework creates a standard of assurance for GPCPs [25]. Core Advanced credentialed pharmacists demonstrate competency in delivering care for patient populations with complex needs, act as autonomous decision-makers in uncertain or evidence-poor contexts and apply advanced clinical assessment and examination techniques adapted to individual patients’ needs [25]. Yet, senior pharmacy leadership may have insufficient understanding of advanced pharmacist practice roles, creating barriers to successful implementation to meet service needs [21, 24, 26, 27]. Furthermore, UK pharmacist education and training reforms direct that from 2026, newly qualified pharmacists will have independent prescribing credentials [28]. However, currently credentialed prescribing pharmacists report low confidence in decision-making [29], with limited access to structured development support, reducing their ability to autonomously manage clinical caseloads [29, 30]. Addressing these challenges is essential to ensuring GPCP prescribers are supported to practice to an advanced level to meet service demands and patient needs [31].

Structured programmes support pharmacists transitioning into expanded clinical roles with greater reassurance and reduced uncertainty [25, 29, 32]. Structured programmes are recommended enablers for developing pharmacists’ professional knowledge, skills and behaviours to autonomously deliver care [22, 23, 29–31, 33–37]. Preceptorship differs from didactic training by aiming to integrate healthcare professionals into practice during transitional career stages through workplace-based learning, supervision, feedback and reflection during routine clinical practice [38]. Preceptorship may support pharmacists to build competence and confidence towards advanced practice [35] although studies prospectively evaluating the impact of preceptorship beyond undergraduate-to-newly qualified period are limited [35]. Therefore, NHS Greater Glasgow and Clyde (NHSGGC) developed and evaluated a 12-week preceptorship programme to support GPCPs working towards advanced practice and developing patient-facing primary care roles.

### Aim

To evaluate the impact of a preceptorship programme for GPCPs on self-assessed training needs, competence and confidence within routine primary care practice.

## Method

### Study design

A prospective repeated-measures survey and multiple medication review activity analysis was conducted in one NHS health board, January 2024 to March 2025. Reported following the Checklist for Reporting of Survey Studies (CROSS) [39].

### Setting

The UK NHS is publicly funded and organised through regional health boards in Scotland. NHSGGC provides healthcare services for 1.3 million people within a diverse urban region, containing 234 family medical practices. Services are delivered across eight Health and Social Care Partnerships (HSCPs) integrating community health and social care services.

### Questionnaire development

The questionnaire included 40 questions across two sections: 8 demographics and 32-item Hennessy-Hicks Training Needs Analysis. It was administered electronically via Microsoft Excel® spreadsheet enabling real-time feedback on individual preceptees’ prioritised learning needs while enabling data capture for analysis. Demographics included: age, gender, professional experience, independent prescriber status and NHS Agenda for Change banding [40].

The Hennessy-Hicks is a validated 30-item self-administered, psychometrically robust questionnaire for identifying staff training needs [41, 42] and often adapted for different healthcare settings and professional groups [43, 44]. The Hennessy-Hicks manual supports question modification without invalidation [41]. Respondents self-score task importance and performance (competence) across five superordinate categories: administration; clinical tasks; communication/teamwork; management and supervision and research and audit. Up to 8 questions may be removed or replaced by items of the researcher’s choice and an additional 10 items may be added while retaining questionnaire validity [41]. In this study, eight questions were removed: six research/audit and one each from the management/supervisory and clinical categories. All modifications followed the Hennessy-Hicks manual [41]. Ten questions were added; aligned with Domains 1 and 2 of the RCPharm Core Advanced Pharmacist Curriculum (person-centred care and professional practice, respectively) to evaluate preceptees’ perceived ability to address NHS Scotland’s *National Therapeutic Indicators* [18], creating a 32-item questionnaire (supplementary files) and a new superordinate category defined as *National Therapeutic Indicators*. [41] Preceptees rated each task along a 7-point scale in two ways: task importance to their role (Rating A) and self-perceived task performance (competence) (Rating B) [41]. A self-perceived confidence rating (Rating C), complementing the Hennessy-Hicks importance and performance ratings, was added by the evaluation team. Questionnaire content validity and clarity were confirmed by six primary care pharmacists involved in programme development. Three GPCPs matching the target cohort were purposefully selected to pilot the questionnaire.

### Recruitment and delivery

In 2024, NHSGGC employed 198 GPCPs, with 138 potentially meeting preceptee inclusion criteria: NHSGGC employed; band 6 and 7; patient-facing, enrolled in an RCPharm development portfolio. Previous studies have highlighted that this group had greater training needs compared to senior colleagues [21–23]. Eligible preceptees were identified by managers responsible for workforce deployment and ability to confirm eligibility criteria fulfilment. Managers were not involved in questionnaire completion, data collection or analysis. Questionnaires were returned directly to the evaluation team. Prior preceptorship programme participation was not collected as preceptorship is not routinely embedded in the UK [35]. Control group usage was restricted with service and operational requirements unable to justify withholding preceptorship from otherwise eligible pharmacists. Likewise, as delivery was within a live service, sampling was pragmatic.

**Fig. 1:**
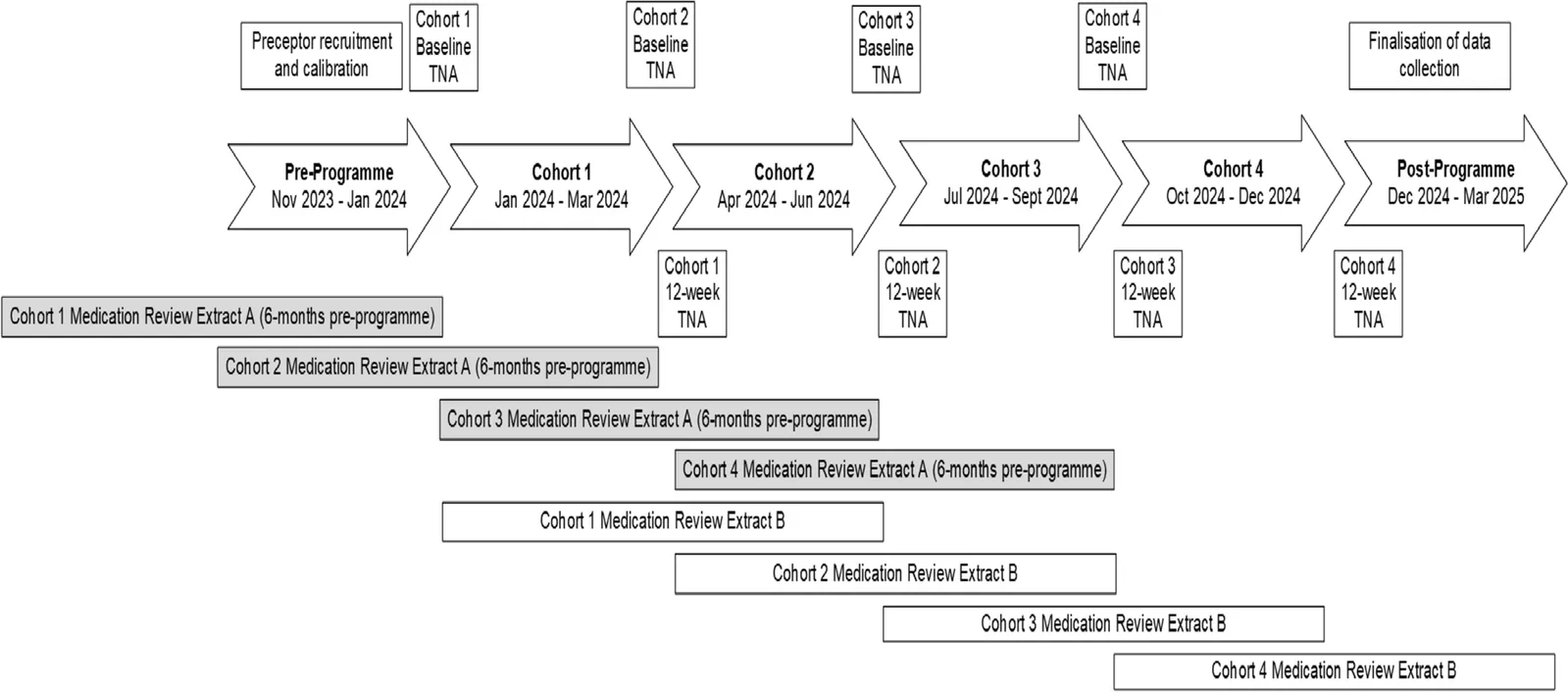
Preceptorship programme development and delivery timeline. *Notes*: TNA: Training needs assessment “Hennessy-Hicks” questionnaire. Extract A: counts of medication reviews 6 months prior to programme commencement. Extract B: counts of medication reviews 6 months post-programme commencement

Eight preceptors meeting predefined criteria: NHSGGC employee; delivering regular autonomous patient-facing clinics; active independent prescribers; experienced as clinical supervisors including completion of national supervision and feedback training modules [45] were recruited. Preceptors attended a mandatory calibration session with programme developers. This included programme expectations, self-assessment of competence and knowledge gaps (supplementary files), and promotion of balanced and consistent preceptee feedback aligned to the RCPharm portfolio [25]. Preceptors were granted protected programme delivery time (1 to 2 days/week), creating capacity to support 96 preceptees, once weekly for 12 weeks during patient-facing clinics, and provided verbal and written feedback (supervised learning events). Four preceptee-cohorts participated during the 12-month evaluation, Fig. 1.

### Data collection

GPCP-preceptees were directly invited to complete the questionnaire by the evaluation lead (TC) via email, to identify training needs, competence and confidence at baseline (pre-programme) and after 12 weeks of preceptorship. Reminder emails were sent one week after the initial email. Both emails contained participant information, consent form, and questionnaire.

Within NHS Scotland, pharmacists undertaking medication reviews for multiple medications (polypharmacy) record completion in the electronic clinical record using Read Code (8B31B) informing national activity measures. Read Codes are a standard hierarchical classification system for recording patient medical information in UK primary care [46]. Counts of 8B31B were used as a routinely recorded proxy-measure of medication review activity. Due to variation in coding practices, this was considered appropriate contextual evidence rather than a measure for inferential comparison of clinical performance. Data were extracted retrospectively from practice electronic clinical records using a pre-existing NHSGGC Microsoft Access® tool routinely used to count unique instances of 8B31B within patient records for individual preceptees delivering reviews. Data were extracted for six months pre-programme (Extract A) and post-programme commencement (Extract B), Fig. 1, and submitted to the evaluation team.

### Data management and analysis

Preceptee-level survey data were pseudonymised and analysed using matched pre- and post-programme responses collated in Excel®. For each participant, priority scores for questionnaire items were calculated by subtracting self-reported performance score (Rating B) from the importance score (Rating A) [41], and interpreted as an unmet training need rather than a measure of objective competence or task frequency. An overall mean priority score per task across all participants was calculated to rank unmet training needs [41]. Overall mean confidence (Rating C) scores were also calculated. Quadrant graphs of aggregated values for the 32 items were developed visually summarising pre- to post-programme training needs prioritisation. Due to clustering effects, the top five priorities were tabulated and graphed for clarity. The Wilcoxon signed-rank test was applied comparing baseline and 12-week questionnaire scores with p-values reported for comparison of pre- and post-programme questionnaire outcomes. Results of testing are included for information purposes, however, as this is a small prospective evaluation, all analyses are hypothesis-generating. Preceptees’ medication review activity data were analysed descriptively with a run chart of the median number of medication reviews and interquartile range (IQR) over time.

### Ethics approval

The West of Scotland Research Ethics Committee was consulted and confirmed that this project was a service evaluation of workforce development not requiring formal ethical approval. Participants were provided with information about the evaluation and consented electronically prior to completing the questionnaire.

## Results

Fifty-three GPCP-preceptees were recruited with participant dropout due to employment changes or ill-health, resulting in 51 participants completing the 12-week programme. Median age was 34 (range 24–57) years; 43 (84%) were female, median years qualified 11 (range 1–33) and 3 (range 0–10) years’ experience working in general practice settings. The majority (70%, n = 36) were active independent prescribers, Table 1.

**Table 1:** Pharmacist participant demographics and characteristics (n = 51)

	n
Age, median [minimum–maximum]	34 [24 to 57]
Gender, identify as female (%)	43 (84.3)
Years qualified, median [minimum–maximum]	11 [1 to 33]
Years working in general practice setting, median [minimum–maximum]	3 [0 to 10]
Years working hospital setting, median [minimum–maximum]	0 [0 to 10]
Years working community pharmacy setting, median [minimum–maximum]	6 [0 to 27]
*NHS ‘Agenda for change’ professional banding*	
Band 6 (%)	3 (6)
Band 7 (%)	48 (94)
*Prescribing status (%)*	
Qualified independent prescriber and actively prescribing (%)	36 (70)
Qualified independent prescriber NOT actively prescribing (%)	2 (4)
Currently studying to become an independent prescriber (%)	10 (20)
Non-prescriber (%)	3 (6)

### Hennessy-hicks training needs analysis

**Fig. 2:**
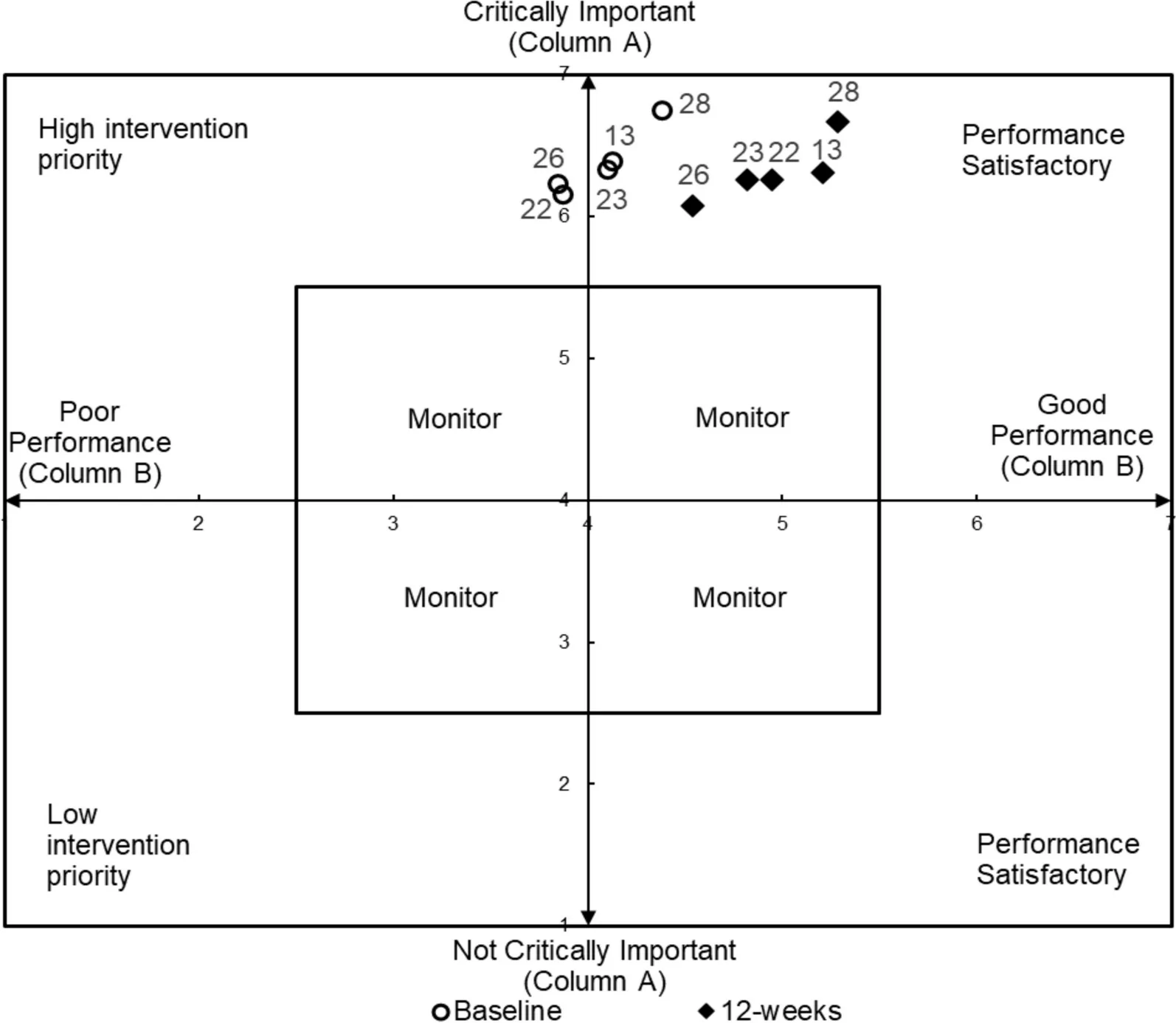
Pre- to post-programme changes in 5 highest ranking unmet training needs^*b*^. ^*b*^Mean task performance versus importance (n = 39 preceptees). Baseline (open circles) to after 12-weeks (diamonds) of preceptorship programme completion

Fifty-one preceptees completed the programme, 39 (76%) of whom completed both pre- and post-programme questionnaires, with matched responses being analysed. Analysis of ranked unmet training need scores revealed the five highest-ranking training needs, in descending order: assessment and management of mental health (Q26) and chronic pain (Q28) (aligned to *National Therapeutic Indicators*); managing conflict, hostility, trauma and hesitancy (Q22); conducting biopsychosocial assessment (Q13) and assessing capacity and facilitating patient-informed healthcare decisions (Q23), Table 2 and Fig. 2. Two tasks, Q26 (Undertake reviews and optimise treatment for mental health) and Q22 (Managing hostility and conflict), appeared in the *High priority intervention* quadrant, Fig. 2.

**Table 2:** Top 5 ranked baseline priority unmet training needs and change in unmet training need scores after 12 weeks of preceptorship (n = 39)

Q no	Task	Priority score^a^			Wilcoxon signed-rank		
		Baseline	After 12 weeks	Z		*p*	*r*
26	Undertake reviews and optimise treatment appropriately for Mental Health conditions	2.38	1.54	− 3.899		< 0.001	− 0.441
28	Undertake reviews and optimise treatment appropriately for Chronic Pain	2.36	1.38	− 4.818		< 0.001	− 0.546
22	Managing hostility and significant conflict, overcoming resistance or hesitancy, delivering distressing or upsetting information, and/or engaging with people who are distressed, or suffering from acute severe physical (e.g. pain), mental or emotional illness or trauma, appropriately	2.28	1.31	− 4.275		< 0.001	− 0.484
13	Evaluating patients physical, psychological and social needs (i.e. bio-psycho-social assessment—holistically assessing biological, psychological, social causes of signs/symptoms)	2.26	1.10	− 4.779		< 0.001	− 0.541
23	Assessing the capacity of individuals to make decisions with respect to the care being delivered by the pharmacist and effectively manage situations where capacity is lacking, recognise people who may need support in articulating their healthcare needs and taking steps to enable this	2.23	1.44	− 3.955		< 0.001	− 0.448

^a^ Priority score calculated as task importance rating minus self-perceived performance (competence) rating. A score reduction pre- to post- preceptorship indicates a smaller gap between self-perceived task importance and self-perceived task performance (competence) and therefore a reduction in self-reported unmet training need for that task. Scores do not represent task frequency or objective clinical performance

Pre- to post-programme analysis identified reductions in priority unmet training need scores across 32 questionnaire items (supplementary files). For the five highest-ranked baseline priority tasks, paired analysis showed significant (*p* < 0.001) reductions in priority scores after 12 weeks; Table 2. Four items (Q26, Q28, Q22, Q23) remained within the top five ranked tasks with a right shift towards the *Performance satisfactory* quadrant (Fig. 2). One task (Q13) shifted to rank 11. For all tasks, there was convergence in importance (Rating A) and performance (Rating B) scores with the largest descriptive change seen in self-reported performance.

**Fig. 3:**
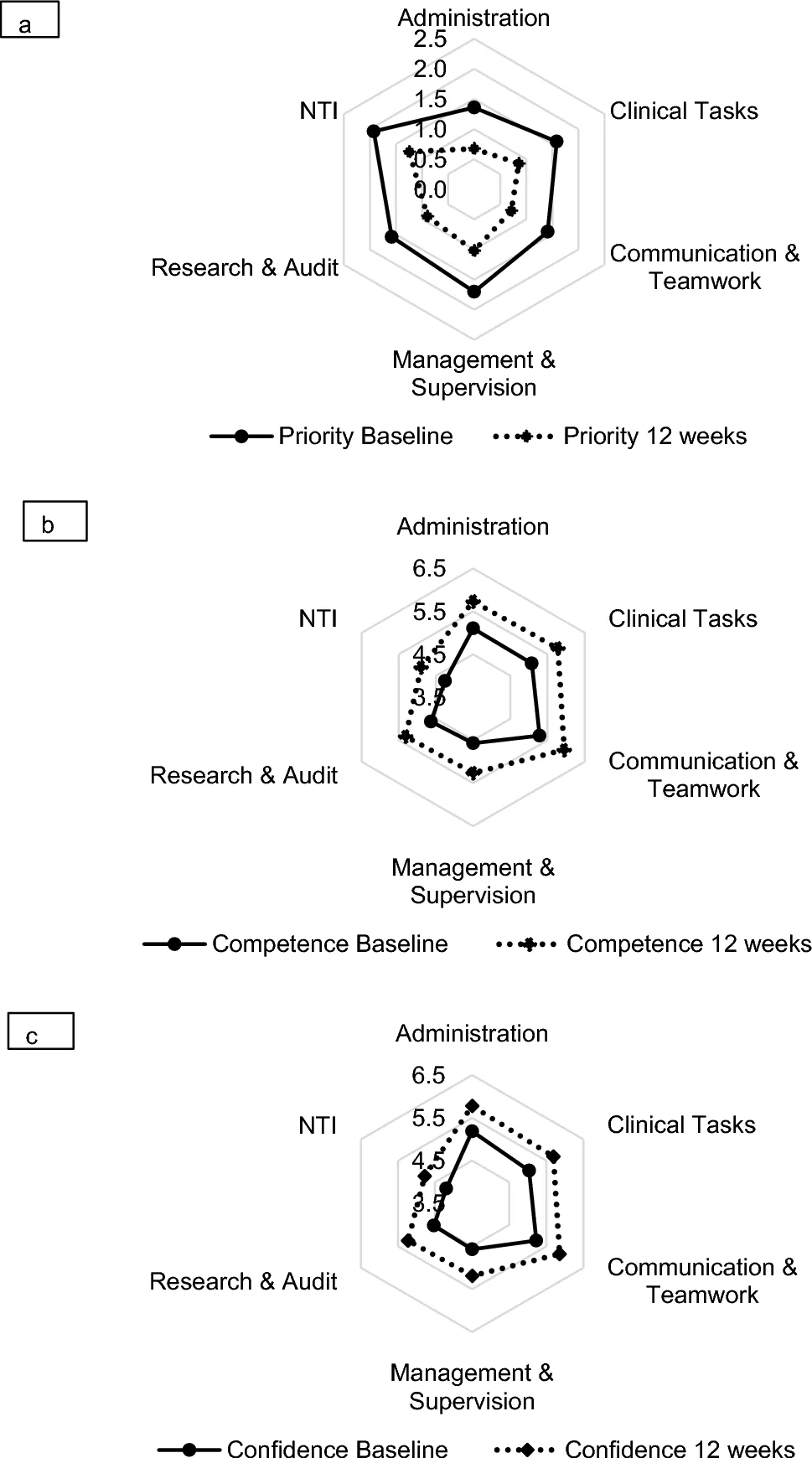
Pre- to post-programme self-reported change in superordinate category scores^*c*^ (n = 39). ^*c*^Panel a. Change to priority learning needs across each superordinate category, lower scores indicate lower self-reported training needs; panel b. Change to self-reported competence across each superordinate category, higher scores equate greater self-reported competence; panel c. Change to self-reported confidence across each superordinate category, higher scores equate greater self-reported confidence. NTI = *National Therapeutic Indicators*

Descriptive superordinate category summaries indicated lower unmet training need scores and higher self-reported competence and confidence after 12 weeks. (Fig. 3). Within the priority training needs (Fig. 3, Panel a) all superordinate categories indicated similar improvements with the *Clinical* category demonstrating the numerically largest improvement. The *National Therapeutic Indicator* category remained an outlying spike matching trends seen within the individual top 5 training need priorities. The self-reported competence and confidence radar charts (Fig. 3, Panel b and c respectively) indicate improvements in competence and confidence in performing tasks across all superordinate categories while showing that the priority training needs lie within the *National Therapeutic Indicator* category.

### Medication review activity

**Fig. 4:**
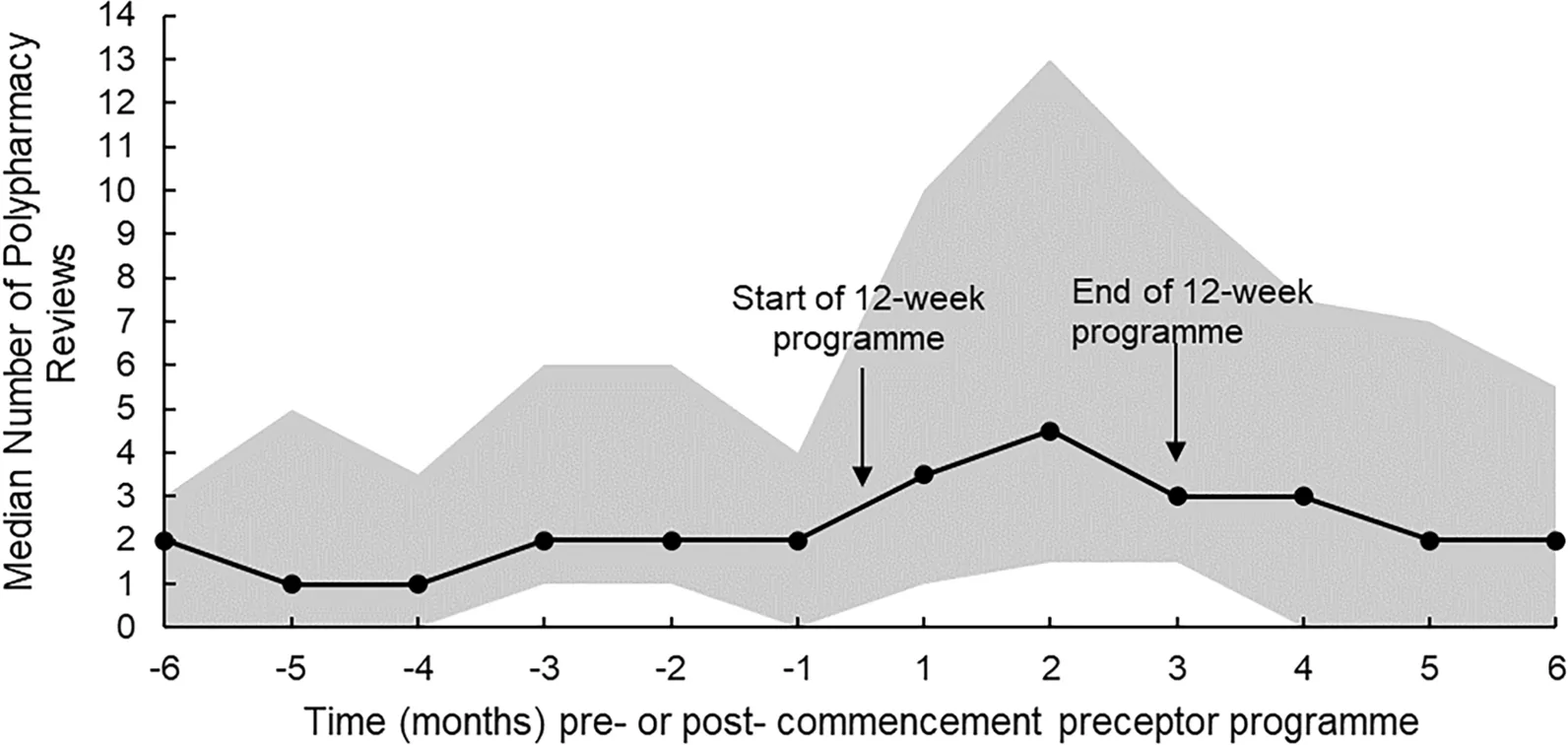
Median^d^ number of polypharmacy medication reviews pre- and post-preceptorship programme ^d^ grey shaded band indicates the interquartile range (IQR) (n=37 preceptees)

Baseline medication review data (Extract A) was available for 46 (90%) preceptees, as five preceptees were unable to return Extract A due to IT compatibility issues. Nine preceptees did not return the post-preceptorship data (Extract B) and were therefore excluded from the activity analysis. Extract A for pharmacists missing Extract B was reviewed descriptively and did not differ compared to those who returned Extract A and B. Therefore, unmatched extracts were excluded from analysis resulting in 37 (73%) preceptees’ data being analysed. Recorded medication review activity per preceptee was variable and was summarised descriptively (Fig. 4). Median monthly per preceptee reviews increased from 2 (IQR 0–4) pre-preceptorship to 4 (IQR 1–10) during the programme, falling to 2 (IQR 0–7) in the 3 months post-programme completion. These data describe recorded activity volume only.

## Discussion

This pharmacist preceptorship programme observed reductions in self-reported unmet training need scores. However, the persistence of specific high-ranked training needs (hostility and conflict management, biopsychosocial assessment and capacity-related decisions, mental health and chronic pain) may suggest gaps in the consultation, clinical reasoning and risk management skills required by pharmacists developing advanced patient-facing primary care roles [25]. Findings should be interpreted as a self-perceived developmental change rather than objective performance improvement. Medication review activity counts increased during preceptorship but were not sustained. Future programme design must consider the consistency and accessibility of organisational infrastructure including follow-on supervision, performance management and assurance processes that are likely needed to sustain and further develop practice.

Primary care workforce pressures including increasing caseloads and recruitment difficulties [5, 47, 48] highlight the need for GPCP-led models of care underpinned with professional support structures [36]. Pharmacists entering and developing primary care roles experience uncertainty and a lack of support to develop a generalist scope of practice [21–24, 26], which may affect preparedness to perform advanced patient-facing roles [22, 30]. The RCPharm Core Advanced Curriculum describes entry-level advanced pharmacist practice and recommends supervisory support [25]. Likewise, literature describes potential influences on pharmacist advanced practice behaviours and challenges in encouraging autonomous practice which may be mitigated using preceptorship programmes [31, 37]. While preceptorship provides structured support, the integration of all strands of clinical supervision, including peer review, ongoing reflection and managerial supervision offers an integrated approach to address professional isolation, promote pharmacist confidence and support safer practice in an increasingly complex healthcare environment [36, 49]. Therefore, formal and consistent, structured clinical supervision models are necessary to develop pharmacist practice [22, 23, 29–31, 33–37]. This aligns with national priorities to develop an adaptable and confident pharmacy workforce capable of delivering high-quality, person-centred and cost-effective pharmaceutical care in primary care [50, 51].

### Strengths and limitations

One strength was inclusion of the validated Hennessy-Hicks tool, adding to the robustness of findings, as pharmacy services evaluations often lack validated tool use [23]. However, pragmatic recruitment and small sample size may limit meaningful observed differences, and initial power calculations were not carried out due to the lack of studies assessing preceptorship in primary care settings [35], yet these findings may help to inform future studies. Another strength was that baseline training needs were identified and re-evaluated after 12 weeks of preceptorship demonstrating the potential impact of the preceptorship model, unlike previous single-timepoint pharmacy studies that identified training needs without implementing an intervention [22, 23, 31]. Multiple GPCP-preceptors delivered the programme across four preceptee cohorts therefore reducing bias from using a single preceptor, improving external validity and likelihood of successful implementation within real-world practice. However, despite preceptor calibration, individual differences in supervisory style may have influenced outcomes and was not studied. Lastly, conducting the programme within one large health board with a shared management structure, priorities and role expectations [7], supported a consistent implementation strategy. The reliance on self-reported measures may not reflect objective performance improvement. Results may be influenced by social desirability bias where preceptees wished to appear improved compared to baseline therefore providing improved scoring due to belief this was expected [52, 53]. Although adaptions were supported by the questionnaire manual, the internal consistency of the modified Hennessy-Hicks/NTI domains and the locally developed confidence scale was not formally evaluated. Similarly, increased medication review activity during preceptorship may demonstrate a “*Hawthorne Effect”* with preceptees modifying usual practice due to preceptor presence [54]. The inclusion of a control group may have strengthened interpretation of findings. Manager-led recruitment may be influenced by perceived developmental readiness, service need or participant motivation. The variety of participants, while reflective of current workforce, limits sub-group analysis to understand impacts of past experience or qualifications. Prior exposure to preceptorship, although not widely available beyond undergraduate-newly qualified level [35], was not collected but may have influenced baseline perceived training needs. Using routine Read coded activity as a proxy-marker may have been less effective where pharmacists did not consistently record completed reviews because of competing service pressures. Medication review activity tracking was restricted to three months following programme completion limiting the assessment of the sustainability of the intervention’s effect. Although conducted in one Scottish health board, the service context of patient-facing medication management and medication review is not unique within UK family medical pharmacy practice. Adaptation may be required for healthcare systems where pharmacist scope of practice, workforce models or service priorities differ, possibly informing development of similar preceptorship approaches.

### Further research

The persistence of mental health as a high priority training need is consistent with previous research [23] and is clinically and socially important for further examination through qualitative methods. Qualitative evaluation of preceptee and preceptor experiences of preceptorship programmes including examination of the practical resource implications and support structures required to sustain developmental change will support refinement of future interventions. Evaluation of the potential impact on patient outcomes, medication safety and prescribing quality are necessary. Developing consensus on the scope and expectations of advanced pharmacist practice roles within the primary care setting will reduce ambiguity and facilitate shared understanding between pharmacists, other healthcare professionals and service managers, enabling more effective workforce planning and appropriate utilisation of pharmacists’ skills to better support service delivery and patient care.

## Conclusion

This study describes the changes associated with a structured preceptorship programme for pharmacists working in patient-facing family practice roles. The findings suggest that preceptorship may support improvement in pharmacist self-perceived confidence, competence and prioritised training needs, highlighting the potential value of embedding preceptorship programmes into routine service delivery. Sustained investment in preceptor roles and structured supervision programmes offer a promising strategy to support workforce development needs and contribute to improving service delivery within primary care.

## Supplementary Information

Below is the link to the electronic supplementary material.

Supplementary file1 (DOCX 38 KB)

## Data Availability

The data generated and analysed during the evaluation are not publicly available as they relate to local NHS workforce and potentially contain identifiable workforce information. De-identified aggregate data may be available from the corresponding author on reasonable request, subject to NHS Greater Glasgow and Clyde information governance requirements and approvals.

## References

[1] Ikpoh M, Marshall M. The workforce crisis in general practice. Br J Gen Pract. 2022;72:204–5. 10.3399/bjgp22X719213. 35483948 PMC11189033

[2] Owen K, Hopkins T, Shortland T, et al. GP retention in the UK: a worsening crisis. Findings from a cross-sectional survey. BMJ Open. 2019;9:e026048. 10.1136/bmjopen-2018-026048. 30814114 PMC6398901

[3] Australian Government Department of Health and Aged Care. Supply and demand study general: practitioners in Australia 2024 [Internet]. Canberra: Australian Government; 2024. Available from https://hwd.health.gov.au/supply-and-demand/gp-supply-demand-study.html. Accessed 24 Jan 2026.

[4] Andrew A. Aotearoa New Zealand general practice workforce crisis: what are our solutions? J Prim Health Care. 2024;16:214–7. 10.1071/HC23178. 38941252

[5] Health and Social Care Committee. The future of general practice: Fourth Report of Session 2022–23. HC113 [Internet]. London: House of Commons; 2022. Available from https://committees.parliament.uk/publications/30383/documents/176291/default/. Accessed 14 Dec 2025

[6] Barnett K, Mercer SW, Norbury M, et al. Epidemiology of multimorbidity and implications for health care, research, and medical education: a cross-sectional study. Lancet. 2012;380:37–43. 10.1016/S0140-6736(12)60240-2. 22579043

[7] Johnson CF, Maskrey M, MacBride-Stewart S, et al. New ways of working releasing general practitioner capacity with pharmacy prescribing support: a cost-consequence analysis. Fam Pract. 2022;39:648–55. 10.1093/fampra/cmab175. 35016210

[8] Tan ECK, Stewart K, Elliott RA, et al. Pharmacist services provided in general practice clinics: a systematic review and meta-analysis. Res Social Adm Pharm. 2014;10:608–22. 10.1016/j.sapharm.2013.08.006. 24161491

[9] Hunt V, Anderson D, Lowrie R, et al. A non-randomised controlled pilot study of clinical pharmacist collaborative intervention for community dwelling patients with COPD. npj Prim Care Respir Med. 2018;28:38. 10.1038/s41533-018-0105-7. 30305634 PMC6180130

[10] Leong SL, Teoh SL, Fun WH, et al. Task shifting in primary care to tackle healthcare worker shortages: an umbrella review. Eur J Gen Pract. 2021;27:198–210. 10.1080/13814788.2021.1954616. 34334095 PMC8330741

[11] Sudeshika T, Naunton M, Deeks LS, et al. General practice pharmacists in Australia: a systematic review. PLoS ONE. 2021;16:e0258674. 10.1371/journal.pone.0258674. 34648595 PMC8516208

[12] Croke A, Cardwell K, Clyne B, et al. The effectiveness and cost of integrating pharmacists within general practice to optimize prescribing and health outcomes in primary care patients with polypharmacy: a systematic review. BMC Prim Care. 2023;24:41. 10.1186/s12875-022-01952-z. 36747132 PMC9901090

[13] Johnson CF, Ingram F, Thomson F, et al. General practice pharmacist-led antipsychotic physical health monitoring: a prospective intervention scoping study. Fam Pract. 2024;41:41–9. 10.1093/fampra/cmad120. 38180874

[14] Royal Pharmaceutical Society, Royal College of General Practitioners. Joint policy statement on general practice based pharmacists [Internet]. London: Royal Pharmaceutical Society; 2016. Available from https://rcpharm.wpenginepowered.com/wp-content/uploads/2026/04/rcgps-rps-joint-statement-on-general-practice-based-pharmacists-final-full-version-Scotland.pdf. Accessed 21 Aug 2025.

[15] NHS England. More than 400 pharmacists to be recruited to GP surgeries by next year. 2015. Available from: https://www.england.nhs.uk/2015/11/pharmacists-recruited. Accessed 1 Mar 2026. 10.1136/bmj.h6167 26572158

[16] NHS Education for Scotland. Pharmacy Workforce Report 2023 [Internet]. Edinburgh: NHS Education for Scotland; 2024. Available from https://turasdata.nes.nhs.scot/data-and-reports/other-workforce-statistics/dental-and-pharmacy-workforce-reports/all-other-publications/pharmacy-workforce-2023/?pageid=12501. Accessed 27 Aug 2025.

[17] Scottish Government. The 2018 General Medical Services Contract in Scotland [Internet]. Edinburgh: Scottish Government; 2017. Available from https://www.gov.scot/publications/gms-contract-scotland/. Accessed 27 Aug 2025.

[18] Scottish Government. Effective prescribing and therapeutics. Available from https://www.gov.scot/policies/health-improvement/effective-prescribing-and-therapeutics/. Accessed 1 Mar 2026.

[19] Matheson C, Reid F, Stewart F, et al. Development of an education and support framework for pharmacists working in GP practice. Int J Pharm Pract. 2020;28:191–9. 10.1111/ijpp.12610. 32125750

[20] Weir N, Newham R, Dunlop E, et al. The impact of the COVID-19 pandemic on pharmacy personnel in primary care. Prim Health Care Res Dev. 2022;23:e56. 10.1017/S1463423622000445. 36093791 PMC9472301

[21] Campbell I, Harrison H, Kurdi A. A qualitative study exploring the challenges and enablers of pharmacists with a recent background in community pharmacy transitioning into primary care. Int J Clin Pharm. 2024;46:704–13. 10.1007/s11096-024-01710-4. 38478211

[22] Clarke C, Tennant S, Greenlaw N, et al. The cardiology training needs of general practice-based pharmacists. Int J Pharm Pract. 2021;29:245–51. 10.1093/ijpp/riaa016. 33793792

[23] Johnson CF, Earle-Payne K. Identifying mental health training needs of general practice pharmacy workforce to advance practice: a training needs analysis survey. Int J Clin Pharm. 2022;44:1454–63. 10.1007/s11096-022-01486-5. 36282414

[24] Akhtar N, Hasan SS, Babar ZUD. Evaluation of general practice pharmacists’ role by key stakeholders in England and Australia. J Pharm Health Serv Res. 2022;13:31–40. 10.1093/jphsr/rmac002.

[25] Royal Pharmaceutical Society. Core advanced pharmacist curriculum [Internet]. London: Royal Pharmaceutical Society; 2022. Available from https://www.rcpharm.org/education/core-advanced-pharmacist-curriculum/. Accessed 27 May 2026.

[26] Bartlett S, Bullock A, Spittle K. I thought it would be a very clearly defined role and actually it wasn’t”: a qualitative study of transition training for pharmacists moving into general practice settings in Wales. BMJ Open. 2021;11:e051684. 10.1136/bmjopen-2021-051684. 34697116 PMC8547357

[27] McLean MA, Forsyth P, Boyter AC. Pharmacists’ views on barriers and enablers to the implementation of advanced pharmacist prescribing in Scotland: a qualitative study using normalisation process theory. Int J Clin Pharm. 2025. 10.1007/s11096-025-02021-y. 41051718 PMC12992344

[28] General Pharmaceutical Council. Guidance to support the implementation of the standards for the education and training of pharmacist independent prescribers [Internet]. London: General Pharmaceutical Council; 2022. Available from https://www.pharmacyregulation.org/students-and-trainees/pharmacist-education-and-training/independent-prescriber-education-and-training. Accessed 13 Sep 2025.

[29] Bailey G, Dunlop E, Forsyth P. A qualitative exploration of the enablers and barriers to the provision of outpatient clinics by hospital pharmacists. Int J Clin Pharm. 2022;44:1013–27. 10.1007/s11096-022-01435-2. 35799036 PMC9263044

[30] Alexander L, Rajiah K, Courtenay A, et al. Confidence, barriers, and role identity of general practice independent pharmacist prescribers in Northern Ireland. Healthcare (Basel). 2025;13(8):933. 10.3390/healthcare13080933. 40281881 PMC12027101

[31] Rushworth GF, Jebara T, Tonna AP, et al. General practice pharmacists’ implementation of advanced clinical assessment skills: a qualitative study of behavioural determinants. Int J Clin Pharm. 2022;44:1417–24. 10.1007/s11096-022-01484-7. 36214937 PMC9718702

[32] Deasy E, Seoighe A, Ryan C, et al. Pharmacy stakeholders’ views and experiences of the credentialing of advanced or specialist pharmacist practice: a mixed methods systematic review. Explor Res Clin Soc Pharm. 2024;16:100522. 10.1016/j.rcsop.2024.100522. 39624069 PMC11609238

[33] Nevers W, Ratcheva A, Boutin K, et al. Barriers to and enablers of implementation of high-value interventions by renal pharmacists: a qualitative study informed by the theoretical domains framework. Can J Hosp Pharm. 2020;73(3):177–85. 10.4212/cjhp.v73i3.2995. 32616943 PMC7308154

[34] Choudhary T, Newham R. The advanced clinical practice pharmacy role and its implementation to practice in England. Pharm Educ. 2020;20:215–24. 10.46542/pe.2020.201.215224.

[35] McLean MA, Forsyth P, Dunlop E, et al. Developing a pharmacist preceptorship programme to support UK advanced level practice: a consensus study. Int J Clin Pharm. 2025;47:1248–60. 10.1007/s11096-025-01909-z. 40238030 PMC12432034

[36] Rushworth GF, Forsyth P, Radley A, et al. A pharmacist clinician model as part of a collaborative clinical workforce: a philosophical critique. Res Social Adm Pharm. 2024;20:918–25. 10.1016/j.sapharm.2024.06.006. 38902135

[37] Forsyth P, Rushworth GF. Advanced pharmacist practice: where is the United Kingdom in pursuit of this ‘Brave New World’? Int J Clin Pharm. 2021;43:1426–30. 10.1007/s11096-021-01276-5. 33991288

[38] National Health Service. National Preceptorship Framework for Nursing. [Internet] London: National Health Service; 2022. Available from https://www.england.nhs.uk/long-read/national-preceptorship-framework-for-nursing/. Accessed 13 Sep 2025.

[39] Sharma A, Minh Duc NT, Luu Lam Thang T, et al. A consensus-based checklist for reporting of survey studies (CROSS). J Gen Intern Med. 2021;36:3179–87. 10.1007/s11606-021-06737-1. 33886027 PMC8481359

[40] NHS Employers. NHS Terms and Conditions of Service Handbook [Internet] London: NHS Employers; 2021. Available from https://www.nhsemployers.org/publications/tchandbook. Accessed 18 Aug 2026.

[41] Hicks C, Hennessy D. Hennessy-Hicks Training Needs Analysis Questionnaire and Manual [Internet]. Birmingham: University of Birmingham; 2011. Available from https://epapers.bham.ac.uk/3453/10/HenneseyToolkit.pdf. Accessed 14 Nov 2024.

[42] Hicks C, Hennessy D, Barwell F. Development of a psychometrically valid training needs analysis instrument for use with primary health care teams. Health Serv Manage Res. 1996;9:262–72. 10.1177/095148489600900406. 10162523

[43] Hicks C, Hennessy D. The use of a customized training needs analysis tool for nurse practitioner development. J Adv Nurs. 1997;26:389–98. 10.1046/j.1365-2648.1997.1997026389.x. 9292375

[44] Markaki A, Malhotra S, Billings R, et al. Training needs assessment: tool utilization and global impact. BMC Med Educ. 2021;21:281. 10.1186/s12909-021-02748-y. 34059018 PMC8167940

[45] NHS Education for Scotland. Supervision | Turas | Learn [Internet]. Edinburgh: NHS Education for Scotland. Available from: https://learn.nes.nhs.scot/67639. Accessed 1 Mar 2026.

[46] Chisholm J. The Read clinical classification. BMJ. 1990;300:1092. 10.1136/bmj.300.6732.1092. 2344534 PMC1662793

[47] Hobbs FDR, Bankhead C, Mukhtar T, et al. Clinical workload in UK primary care: a retrospective analysis of 100 million consultations in England, 2007-14. Lancet. 2007;387:2323–30. 10.1016/S0140-6736(16)00620-6. PMC4899422 27059888

[48] Doran N, Fox F, Rodham K, et al. Lost to the NHS: a mixed methods study of why GPs leave practice early in England. Br J Gen Pract. 2016;66:e128–35. 10.3399/bjgp16X683425. 26740606 PMC4723211

[49] Forsyth P, Maguire B, Carey J, et al. Alienation and/or anomie in pharmacists: a systematic review and narrative synthesis of the international literature. Res Social Adm Pharm. 2025. 10.1016/j.sapharm.2025.01.017. 39971637

[50] Scottish Government. Achieving excellence in pharmaceutical care: a strategy for Scotland [Internet]. Edinburgh: Scottish Government; 2017. Available from https://www.gov.scot/publications/achieving-excellence-pharmaceutical-care-strategy-scotland/. Accessed 10 Jun 2025.

[51] Scottish Government. NHS Recovery Plan 2021–2026 [Internet]. Edinburgh: Scottish Government; 2021. Available from https://www.gov.scot/binaries/content/documents/govscot/publications/strategy-plan/2021/08/nhs-recovery-plan/documents/nhs-recovery-plan-2021-2026/nhs-recovery-plan-2021-2026/govscot:document/nhs-recovery-plan-2021-2026.pdf. Accessed 10 Jun 2025.

[52] Crowne DP, Marlowe D. A new scale of social desirability independent of psychopathology. J Consult Psychol. 1960;24:349–54. 10.1037/h0047358. 13813058

[53] Orne MT. On the social psychology of the psychological experiment: with particular reference to demand characteristics and their implications. Am Psychol. 1962;17:776–83. 10.1037/H0043424.

[54] Wickstrom G, Bendix T. The, “Hawthorne effect” - what did the original Hawthorne studies actually show? Scand J Work Environ Health. 2000;26:363–7. 10.5271/sjweh.555. 10994804

