# Supplementary material for: Developing pharmacist practice through a pilot family practice preceptorship programme: a prospective quantitative pre–post evaluation
Source: Int J Clin Pharm. 2026 Aug 6;48(5):2128–38. doi: 10.1007/s11096-026-02198-w (PMC13569539; doi:10.1007/s11096-026-02198-w)
Supplement: Supplementary file 1 [file 11096_2026_2198_MOESM1_ESM.docx]

**Developing Pharmacist Practice Through a Pilot Family Practice Preceptorship Programme: A Prospective Quantitative Pre–Post Evaluation**

# **Author Details**

Tamara M Cairney^1^, MSc, MPharm, BSc (Hons), IP

Advanced Pharmacist, Primary Care

ORCID ID: http://orcid.org/0009-0007-7685-4052

Corresponding author email: tamara.cairney2@nhs.scot

Heather Harrison^1^, MSc, PGDip, BSc (Hons), IP

Senior Prescribing Advisor

Mairi-Anne McLean^1^ PG Dip, BSc (Hons), IP

Senior Prescribing Advisor

ORCID ID: http://orcid.org/0009-0004-5054-4230

Tania Ramos^2^ PhD, MPharm, Cert Adv Clin Pharm, IP

Senior Clinical Pharmacist, Primary Care

ORCID ID https://orcid.org/0009-0002-8055-6502

David Kernaghan^3^ PhD, MSc, BA (Hons)

Research Fellow

ORCID ID: https://orcid.org/0000-0003-0798-4885

Chris F Johnson^1^, PhD, MRes, MSc, BSc (Hons), Cert Psych Pharm, IP, RPS (Consultant Credentialed)

NHS Research Scotland Career Researcher Fellow

Advanced General Practice and Specialist Mental Health Pharmacist

ORCID ID: http://orcid.org/0000-0002-6006-6605

^1^Pharmacy Services, NHS Greater Glasgow and Clyde, Clarkston Court, 56 Busby Road, Glasgow G76 7AT, Scotland, UK

^2^Pharmacy Services, Glasgow City Health and Social Care Partnership (North East Locality), NHS Greater Glasgow and Clyde, Glasgow G69 6GA, UK

^3^School of Health in Social Science, Teviot Place, University of Edinburgh, EH8 9AG

**Supplementary Files – S1: Full Hennessy-Hicks Training Needs Analysis Survey**

| Question | Learning Outcome | How important is this activity to the successful performance of your job?  (Rate 1-7. 1 = not at all important, 7 = very important) | How well do you consider that you currently perform this activity?  (Rate 1-7. 1 = not well, 7 = very well) | How confident do you feel about carrying out this activity?    (Rate 1-7. 1 = not very confident, 7 = very confident)" |
| --- | --- | --- | --- | --- |
| 1 | Establishing relationship with patients, applying patient centred approach – ‘What matters to me’. |  |  |  |
| 2 | Concisely recording medication reviews in clinical notes: issue, action, plan and follow up. |  |  |  |
| 3 | Assessing and applying clinical guidelines (NHSGGC, SIGN, NICE, etc) to patient care. |  |  |  |
| 4 | Communicating effectively with primary and secondary care colleagues regarding multidisciplinary collaborative patient care. |  |  |  |
| 5 | Applying evidence-informed medicine; tailoring pharmacological treatment in areas of clinical uncertainty (i.e deviating from guidelines) |  |  |  |
| 6 | Communicate face to face with patients by effectively adapting verbal and non-verbal communication in challenging situations; demonstrates empathy, values the other person’s point of view to achieve a suitable outcome. |  |  |  |
| 7 | Monitoring medicines safety; acts appropriately and proportionately to minimise their negative effects impact. |  |  |  |
| 8 | Reviews and audits the impact of their practice as part of the service to identify learning and/or alter your scope of practice. |  |  |  |
| 9 | Providing feedback to general practice colleagues for individual patients with complex health needs. |  |  |  |
| 10 | Giving information to patients and/or carer on the risks and benefits of medicines to treat a range of conditions. |  |  |  |
| 11 | Demonstrates ability to identify health inequalities, engages and facilitates individual’s care to reduce such inequities |  |  |  |
| 12 | Planning and organising individual patient’s care (e.g. follow up, discussion with GP, managing risk, uncertainty) |  |  |  |
| 13 | Evaluating patients physical, psychological and social needs (i.e. bio-psycho-social assessment - holistically assessing biological, psychological, social causes of signs/symptoms) |  |  |  |
| 14 | Organising your own time efficiently and effectively to support people with pharmaceutical care needs. |  |  |  |
| 15 | Assessing and addressing environmental impact of medicines e.g. inhalers. |  |  |  |
| 16 | Summarising audit findings and agreeing actions with your general practice(s). |  |  |  |
| 17 | Undertaking health promotion (e.g. smoking cessation, counselling on wellbeing, self-care, sign posting to local services, online resources, etc.) |  |  |  |
| 18 | Assess cost-effectiveness of drug therapy for the treatment of common diseases/disorders |  |  |  |
| 19 | Assessing patients’ clinical needs e.g. have therapeutic objectives been met? |  |  |  |
| 20 | Working as member of the general practice team supporting people with physical and mental health needs |  |  |  |
| 21 | Undertaking medication reviews and optimising treatment appropriately to meet patient’s needs |  |  |  |
| 22 | Managing hostility and significant conflict, overcoming resistance or hesitancy, delivering distressing or upsetting information, and/or engaging with people who are distressed, or suffering from acute severe physical (e.g. pain), mental or emotional illness or trauma, appropriately. |  |  |  |
| 23 | Assessing the capacity of individuals to make decisions with respect to the care being delivered by the pharmacist and effectively manage situations where capacity is lacking, recognise people who may need support in articulating their healthcare needs and taking steps to enable this |  |  |  |
| Undertaking reviews and optimising treatment appropriately to meet patient’s needs for the following therapeutic areas: | | | | |
| 24 | Type 2 Diabetes |  |  |  |
| 25 | Cardiology (includes hypertension, heart failure, atrial fibrillation + IHD) |  |  |  |
| 26 | Mental Health (includes depression, anxiety, bipolar affective disorder, schizophrenia + psychosis, ADHD |  |  |  |
| 27 | Dependence, harm reduction and/or withdrawal (includes alcohol, benzodiazepines, z-hypnotics and opioids) |  |  |  |
| 28 | Chronic Pain |  |  |  |
| 29 | Polypharmacy and frailty |  |  |  |
| 30 | Respiratory (includes asthma + COPD) |  |  |  |
| 31 | Women's Health (includes contraception, menopause, endometriosis and PCOS) |  |  |  |
| 32 | Thyroid Disorders |  |  |  |

**Supplementary Files – S2: NHS GGC Preceptor Readiness Checklist**

**Introduction**

Supervision is an essential element of training to be an advanced practitioner. If you have been asked to supervise a trainee advanced practitioner, it is important to ensure you have the experience, knowledge, and expertise to provide good quality supervision. The tables below have been adapted from various sources including the Royal Pharmaceutical Society Designated Prescribing Practitioner Competency Framework Health Education England [Supervision and assessment resources - Advanced Practice (hee.nhs.uk)](https://advanced-practice.hee.nhs.uk/resources/supervision-and-assessment-resources/) and work done locally. Please use the tables below to assess your readiness to take on the role of a preceptor

**Characteristics/ Knowledge and skills/Teaching and training skills**

| Factors suggesting readiness to provide preceptorship | Examples of evidence | Am I ready? | Personal Action Plan |
| --- | --- | --- | --- |
| I am willing to undertake and recognise the value of the role of the preceptor |  |  |  |
| I create a positive learning environment through being approachable and empathetic |  |  |  |
| I provide objective supervision and assessment and display professional integrity |  |  |  |
| I am an active prescriber in a patient facing role with appropriate knowledge and skills |  |  |  |
| I am undertaking (have completed) a recognised advance/ consultant framework or equivalent experience/ qualification |  |  |  |
| I have completed the NHS Education Scotland TURAS modules for educational supervision or equivalent recognised training in supervision and assessment |  |  |  |

**Delivery of Programme**

| Factors suggesting readiness to provide preceptorship | Examples of evidence | Am I ready? | Personal Action Plan |
| --- | --- | --- | --- |
| I will work with the learner to establish their baseline knowledge and skills, and jointly create a development plan for meeting learning outcomes  Note: Encourage preceptees to access local communities of practice   - *Chronic Pain Network – [Link to NHS GGC Chronic Pain Pharmacist Network]* - *Frailty Network - [Link to NHS GGC Frailty Pharmacist Network]* - *Diabetes Network - [Link to NHS GGC Diabetes Pharmacist Network]* - *GGC Core Advanced Network - [Link to NHS GGC Pharmacist Core Advanced Portfolio Network]* |  |  |  |
| I will provide a minimum of 12 SLEs by the end of the 12-week programme  Materials to support SLE completion:   - *Examples from RPS Core Advanced (scroll to page end) -* [Core advanced pharmacist e-portfolio (rpharms.com)](https://www.rpharms.com/development/credentialing/core-advanced-pharmacist-curriculum/core-advanced-pharmacist-e-portfolio) - *Examples from NHS Education Scotland from past General Practice Clinical Pharmacist Programme* - [Supervised Learning Events (SLEs) \| Turas \| Learn (nhs.scot)](https://learn.nes.nhs.scot/61754) - *All RPS Assessment & Credentialling specific webinars are open access*   - *Available here-* [Webinars \| RPS (rpharms.com)](https://www.rpharms.com/resources/webinars) - *NHS Education Scotland support also available here (log in required) -* [Core advanced pharmacist curriculum \| Turas \| Learn (nhs.scot)](https://learn.nes.nhs.scot/62524) |  |  |  |
| I will work in partnership with the learner, other supervisors and line managers to confirm competence of the learner |  |  |  |
| I will meet with and supervise and assess each learner for one session (3.5 hours) a week for 12 weeks programme |  |  |  |
| I understand the advanced practice role my learner is training towards and I am aware of knowledge, skills and behaviours they require to demonstrate competence  Specific primary care examples which may support demonstration of the RPS Core Advanced Framework outcomes are available via NHS Education Scotland (NES), log-in required:   - Domain 1: Person-Centred Care and Collaboration - [link to NES content pages] - Domain 2: Professional Practice - [link to NES content pages] - Domain 3: Leadership and Management - [link to NES content pages] - Domain 4: Education - [link to NES content pages] - Domain 5: Research - [link to NES content pages] |  |  |  |
| I will undertake regular peer review with other preceptors and constructively critique feedback |  |  |  |
| I am willing to participate in evaluation of the programme and the impact of the intervention |  |  |  |
| I understand the service objectives and the role of the preceptor in delivery |  |  |  |

**Learning and Governance**

| Factors suggesting readiness to provide preceptorship | Examples of evidence | Am I ready? | Personal Action Plan |
| --- | --- | --- | --- |
| I will complete any documentation required for evaluation of the programme |  |  |  |
| I am familiar with my role within the wider governance structures and for the processes for escalating concerns and where appropriate engage in the processes |  |  |  |

**Supplementary Files – S3: Baseline and 12-week post-supervision priority learning need scores ranked in order of priority (highest to lowest) at baseline**

| Q No. | Task | Baseline Priority Score Mean (SD) | After 12 weeks Priority Score Mean (SD) |
| --- | --- | --- | --- |
| 26 | Mental Health (includes depression, anxiety, bipolar affective disorder, schizophrenia + psychosis, ADHD | 2.38 (1.25) | 1.54 (1.05) |
| 28 | Chronic Pain | 2.36 (1.35) | 1.38 (0.81) |
| 22 | Managing hostility and significant conflict, overcoming resistance or hesitancy, delivering distressing or upsetting information, and/or engaging with people who are distressed, or suffering from acute severe physical (e.g. pain), mental or emotional illness or trauma, appropriately. | 2.28 (1.21) | 1.31 (0.89) |
| 13 | Evaluating patients physical, psychological and social needs (i.e. bio-psycho-social assessment - holistically assessing biological, psychological, social causes of signs/symptoms) | 2.26 (1.21) | 1.10 (0.99) |
| 23 | Assessing the capacity of individuals to make decisions with respect to the care being delivered by the pharmacist and effectively manage situations where capacity is lacking, recognise people who may need support in articulating their healthcare needs and taking steps to enable this | 2.23 (1.33) | 1.44 (1.10) |
| 5 | Applying evidence-informed medicine; tailoring pharmacological treatment in areas of clinical uncertainty (i.e. deviating from guidelines)? | 2.08 (1.24) | 1.18 (0.88) |
| 27 | Dependence, harm reduction and/or withdrawal (includes alcohol, benzodiazepines, z-hypnotics and opioids) | 2.03 (1.51) | 1.38 (1.14) |
| 8 | Reviews and audits the impact of their practice as part of the service to identify learning and/or alter your scope of practice. | 2.03 (1.78) | 1.31 (1.24) |
| 11 | Demonstrates ability to identify health inequalities, engages and facilitates individual’s care to reduce such inequities | 1.97 (1.04) | 1.05 (0.92) |
| 29 | Polypharmacy and frailty | 1.90 (0.85) | 1.26 (0.85) |
| 25 | Cardiology (includes hypertension, heart failure, atrial fibrillation + IHD) | 1.85 (0.71) | 1.26 (0.88) |
| 17 | Undertaking health promotion (e.g. smoking cessation, counselling on wellbeing, self-care, sign posting to local services, online resources, etc.) | 1.82 (1.00) | 0.79 (1.00) |
| 24 | Type 2 Diabetes | 1.77 (1.53) | 1.10 (1.14) |
| 30 | Respiratory (includes asthma + COPD) | 1.69 (1.51) | 1.18 (1.23) |
| 21 | Undertaking medication reviews and optimising treatment appropriately to meet patient’s needs | 1.69 (1.08) | 0.95 (0.65) |
| 31 | Women's Health (includes contraception, menopause, endometriosis and PCOS) | 1.62 (1.39) | 1.08 (1.26) |
| 14 | Organising your own time efficiently and effectively to support people with pharmaceutical care needs. | 1.54 (1.27) | 1.05 (0.92) |
| 6 | Communicate face to face with patients by effectively adapting verbal and non-verbal communication in challenging situations; demonstrates empathy, values the other person’s point of view to achieve a suitable outcome. | 1.54 (1.05) | 0.59 (0.68) |
| 9 | Providing feedback to general practice colleagues for individual patients with complex health needs. | 1.51 (1.41) | 0.64 (1.29) |
| 10 | Giving information to patients and/or carer on the risks and benefits of medicines to treat a range of conditions. | 1.49 (1.07) | 0.90 (0.82) |
| 1 | Establishing relationship with patients, applying patient centred approach – ‘What matters to me’. | 1.46 (0.94) | 0.62 (0.54) |
| 32 | Thyroid Disorders | 1.44 (1.52) | 0.85 (1.50) |
| 16 | Summarising audit findings and agreeing actions with your general practice(s). | 1.44 (1.45) | 0.77 (1.01) |
| 20 | Working as member of the general practice team supporting people with physical and mental health needs | 1.41 (0.94) | 0.92 (0.74) |
| 19 | Assessing patients’ clinical needs e.g. have therapeutic objectives been met? | 1.38 (0.85) | 0.85 (0.71) |
| 12 | Planning and organising individual patient’s care (e.g. follow up, discussion with GP, managing risk, uncertainty) | 1.28 (0.97) | 0.77 (0.67) |
| 3 | Assessing and applying clinical guidelines (NHSGGC, SIGN, NICE, etc) to patient care. | 1.23 (1.01) | 0.74 (0.82) |
| 2 | Concisely recording medication reviews in clinical notes: issue, action, plan and follow up. | 1.21 (1.22) | 0.56 (0.68) |
| 7 | Monitoring medicines safety; acts appropriately and proportionately to minimise their negative effects impact. | 1.18 (0.97) | 0.77 (0.71) |
| 15 | Assessing and addressing environmental impact of medicines e.g. inhalers. | 1.18 (1.97) | 0.51 (1.47) |
| 4 | Communicating effectively with primary and secondary care colleagues regarding multidisciplinary collaborative patient care. | 1.05 (0.89) | 0.62 (0.85) |
| 18 | Assess cost-effectiveness of drug therapy for the treatment of common diseases/disorders | 0.69 (1.10) | 0.38 (0.91) |
